# Editorial: The calcium-sensing receptor: from physiology to pharmacology

**DOI:** 10.3389/fphys.2023.1225074

**Published:** 2023-06-06

**Authors:** Marianna Ranieri, Martin Schepelmann, Giovanna Valenti, Enikö Kallay, Daniela Riccardi

**Affiliations:** ^1^ Department of Biosciences, Biotechnologies and Environment, University of Bari Aldo Moro, Bari, Italy; ^2^ Intitute for Pathophysiology and Allergy Research, Medical University of Vienna, Vienna, Austria; ^3^ School of Biosciences, Cardiff University, Cardiff, United Kingdom

**Keywords:** calcium-sensing receptor (CaSR), therapeutic target, CaSR signalling, inflammation, monocytes, PGE2, VGSC, temperature sensing

The Calcium-Sensing Receptor (CaSR) is a G protein-coupled receptor which is in many ways unique. Its best characterized role lies in mineral ion homeostasis via regulation of parathyroid hormone (PTH) secretion and calcium reabsorption in the kidneys (Riccardi and Valenti, 2016). In addition, the CaSR is involved in many other physiological processes, such as bone metabolism, neuronal differentiation, contraction and mineral ion homeostasis of/via smooth muscles, and renal water handling. Lastly, disturbances in CaSR expression or function lead to pathological conditions *e.g.*, to inflammation in the airways and the gut, or pulmonary arterial hypertension (Yarova et al., 2015; Schepelmann et al., 2016; Hannan et al., 2018; Elajnaf et al., 2019; Ranieri et al., 2020; Schepelmann et al., 2021; Yarova et al., 2021; Schepelmann et al., 2022). This multiplicity of functions is achieved via ligand-biased signaling whereby CaSR ligands can evoke differential signaling pathways in a cell-specific manner. A fascinating recently identified feature of the CaSR is regulation of its protein expression levels via microRNA (Gong et al., 2012; Ranieri, 2019). The 6 reviews and research articles collected in this Research Topic summarize and expand on our current understanding of CaSR physiology and its linked pharmacological properties.

The role of the CaSR in inflammatory processes is currently of particular interest, as demonstrated by three of the articles in this Research Topic dealing with this subject. Iamartino and Brandi have reviewed several studies on the role of the CaSR in inducing and modulating pro-inflammatory stimuli in various organs and disease settings, namely, in immune cells, immune check-points, in the nervous system, specifically in the context of Alzheimer’s disease, in the respiratory, cardiovascular, and digestive systems, in adipose tissue, the kidneys, in the context of viral infection, etc. They also examined the possibility of treating such afflictions with negative allosteric CaSR modulators, calcilytics. Fittingly, Werner and Wagner have contributed an insightful review emphasizing CaSR-related effects on monocytes in rheumatoid arthritis. The authors propose that in this disease setting, calciprotein particles are formed due to a combination of increased local calcium concentrations and elevated fetuin-A levels. Together with increased extracellular Ca^2+^ levels and elevated levels of pro-inflammatory cytokines, these particles could lead to a “vicious cycle of inflammation and bone destruction” and targeting this vicious circle may provide a new therapeutic avenue against this disease. In an original research article, Gushchina et al. have shown that activation of the CaSR induces the PGE2 pathway in *in vitro* and *in vivo* models of colitis. Interestingly, the observed induction of PGE2 pathway-specific genes was different between the *in vitro* and *in vivo* settings. On the basis of this and previous results, the authors suggested that calcilytics may constitute a novel way of treating inflammatory bowel disease.

The other three articles of this Research Topic deal with specific features and action modes of the CaSR that highlight the versatility of this protein: in an elegant minireview, Centeno et al., described the role of extracellular phosphate and intracellular phosphorylation in inhibiting and modulating CaSR activity. They illustrate how extracellular phosphate modulates PTH secretion via CaSR inhibition. The authors lead through this topic via an explanation of the structure of the CaSR and its binding sites for various ligands and ions, and progress *via* a detailed account of what is known on the complex interaction of CaSR and extracellular phosphate as well as intracellular CaSR phosphorylation and CaSR activity. Intracellular phosphorylation of the CaSR is also a key feature of yet another non-calciotropic role of the CaSR, namely, that of a temperature sensor. In a pivotal study, Brennan et al. demonstrated that the C-terminal domain of the CaSR mediates its temperature-sensing capabilities. Changes in temperature directly influence CaSR induced intracellular Ca^2+^ oscillation frequency. The anterior preoptic nucleus, a key temperature sensing organ, expresses the CaSR. This organ has been linked to a genetic disorder of thermoregulation in patients with autosomal dominant hypocalcemia with hypercalciuria (ADH) type 1, a disease caused by activating mutations in the CaSR gene. As calcilytics are already being tested for the treatment of ADH, they might also prove beneficial in managing infantile febrile convulsions seen in some ADH children.

Finally, Lindner et al., investigated in a research article the CaSR-*independent* role of the positive allosteric CaSR modulator cinacalcet on voltage-gated sodium channels in neocortical neurons via a yet unknown GPCR other than the CaSR. Using electrophysiological approaches, the authors demonstrated that cinacalcet inhibits these channels and thus neuronal action potentials, especially when the channels are in their fast-inactivated state. The authors conclude that cinacalcet could serve as a template molecule for the development of a new class of sodium channel inhibitors. At the same time, due to its action on neuronal activity, cinacalcet may interact with brain function in patients treated with this drug.

To conclude, the up-to-date and state-of the-art understanding of CaSR pathophysiology presented in this Research Topic would be expected to lead to the identification of novel therapeutic strategies for treating a variety of conditions, including inflammatory diseases and thermoregulation.
